# Aerosol generating procedures, dysphagia assessment and COVID‐19: A rapid review

**DOI:** 10.1111/1460-6984.12544

**Published:** 2020-06-01

**Authors:** Lee Bolton, Claire Mills, Sarah Wallace, Marian C. Brady, Royal College of Speech and Language Therapists (RCSLT) COVID‐19 Advisory Group

**Affiliations:** ^1^ Speech & Language Therapy Service Imperial College Healthcare NHS Trust London UK; ^2^ Speech & Language Therapy Department Leeds Teaching Hospitals NHS Trust Leeds UK; ^3^ Academic Unit of Health Economics University of Leeds Leeds UK; ^4^ Speech & Language Therapy Department, Wythenshawe Hospital Manchester University NHS Foundation Trust Manchester UK; ^5^ Nursing, Midwifery and Allied Health Professions Research Unit Glasgow Caledonian University Glasgow UK

## Introduction

In response to members’ significant concerns and their request for an examination of the evidence relating to oropharyngeal dysphagia assessment, aerosol‐generating procedures (AGPs) and associated risk of COVID‐19 infection, the Royal College of Speech and Language Therapists (RCSLT) established a COVID‐19 Advisory Group (see the appendix). The group aimed to review the evidence underpinning the current healthcare policies in relation to AGPs, dysphagia assessment, and risk of transmission of and infection with COVID‐19 in response to urgent clinical information needs.

## Dysphagia assessment

Oropharyngeal dysphagia assessment is highly complex and may comprise a wide spectrum of interventions including, but not limited to, clinical (bedside) swallowing assessment, provision of therapeutic oral care, fibreoptic endoscopic evaluation of swallowing, videofluoroscopy swallowing study and cough reflex testing. In the UK, dysphagia assessment is often conducted by speech and language therapists (SLTs), though internationally (particularly in regions with no access to SLTs), other multidisciplinary team members may be responsible for this aspect of healthcare. Dysphagia screening will often draw on the skills of the wider multidisciplinary team with specialist nurses in acute stroke and stroke rehabilitation settings, for example, undertaking dysphagia screenings while SLTs undertake in depth dysphagia assessment and a more consultative role (Martino *et al*. 2014). Dysphagia assessment occurs in a range of clinical contexts where there are concerns about a patient's swallowing ability, including acute and critical care, outpatient departments, rehabilitation units, and community settings.

## Review methods

The rapid review focused on clinical (bedside) swallowing assessment and the risk of COVID‐19 transmission through aerosol emissions, the likelihood of aerosol emissions during dysphagia assessment and the evidence supporting the identification of the AGPs identified in COVID‐19 healthcare recommendations. While a standard systematic review approach is preferable when establishing an evidence base for a defined intervention, this was not feasible in the context of the COVID‐19 pandemic for several reasons. COVID‐19 is a novel virus, distinct in many ways from other viral respiratory infections such as Severe Acute Respiratory Syndrome (SARS) or Middle East Respiratory Syndrome (MERS) (Wölfel *et al*. 2020). It was first reported in December 2019 in Wuhan, China, a country where the SLT profession is in its infancy. Early evidence specific to COVID‐19 and dysphagia assessment was anticipated to be scarce. The pace of newly emerging COVID‐19 literature and daily updates to national healthcare policies and recommendations resulted in a rapidly changing literature base which made a traditional in‐depth systematic review approach unfeasible. Conducting a wider systematic search with broader inclusion criteria, such as ‘viral transmission’ and ‘infection rates’ and ‘coughing’ alone, for example, generated a greater number of references (but many irrelevant references based on pilot search strategies), take considerably more time to review and evaluate, but generate findings of questionable relevance to COVID‐19 and the purposes of this review.

### Search strategy

Our rapid review methodology identified relevant literature through a narrow search strategy applied to the following databases (Medline, Embase, Global Health via Ovid and CINAHL via HDAS) using search strategies specific to the electronic database, but searching the following keywords (and variations) ‘infection transmission’, ‘infection control’, ‘healthcare workers’ and ‘procedures’ or ‘dysphagia assessment’ and for free‐text terms ‘aerosol generating procedures’ or ‘bio aerosols’ and ‘healthcare workers’ (for a Medline search strategy example, see the Supplementary Materials). Grey literature sources NICE Evidence, Oxford CEBM COVID‐19 Evidence Service and MedRxiv were also searched. We hand‐searched *JAMA*, *The Lancet* and *BMJ* since December 2019, the UK Public Health England and Health Protection Scotland COVID‐19 policy documents and references cited in those documents, and employed backwards citation searching of key papers identified in the search. We also used PubMed and Google search engines and communicated with the members of the RCSLT COVID‐19 Advisory Group and other international experts.

### Inclusion criteria

We included information relating to COVID‐19 and dysphagia assessment, aerosol generation, risk of infection, transmission, coughing and SLTs. We also reviewed the underpinning evidence informing Public Health England and Health Protection Scotland lists of AGPs and risk of viral transmission or infection and considered to what extent SLTs or dysphagia assessment were included in that evidence base.

All authors reviewed the literature, sharing their findings and supporting references with co‐authors electronically. Through an iterative process, the search findings were collated and summarized with queries, clarifications or explanations. Remaining queries or discrepancies were resolved in a final videoconference discussion between all co‐authors. An initial draft of the final review findings was shared with the wider COVID‐19 Advisory Group for review and feedback.

## COVID‐19 and routes of transmission

The World Health Organisation (WHO) recently concluded that, based on the current evidence, transmission of COVID‐19 is primarily through respiratory droplets and contact routes (*Modes of Transmission of Virus Causing COVID‐19: Implications for IPC Precaution Recommendations* 2020). A high viral load has been detected in the saliva of patients with COVID‐19 with viral shedding observed up to 11 days after hospital admission (To *et al*. 2020b) and in a follow‐up study, up to 25 days after symptom onset (To *et al*. 2020a). Viral shedding from throat swabs is reported for a median of 20.0 days (interquartile range (IQR) = 17.0–24.0) *n* = 137 survivors) and up to 37 days following symptom onset or until death (*n* = 54) (Zhou *et al*. 2020). Research suggests that patients with severe COVID‐19, such as those who are critically ill, have a higher viral load and shed the virus for longer (Liu *et al*. 2020). Emission of respiratory droplets has been acknowledged as an important route of COVID‐19 transmission (*Guidance: Transmission Characteristics and Principles of Infection Prevention and Control* 2020, To *et al*. 2020a).

## COVID‐19 transmission and aerosols

International and national COVID‐19 policy and practice recommendations consistently highlight the emission of very small droplets (aerosols) from COVID‐19 positive patients as increasing the risk of airborne transmission (*Infection Prevention and Control and Preparedness for COVID‐19 in Healthcare Settings. Second Update—31 March 2020* 2020, *COVID‐19 Personal Protective Equipment (PPE)* 2020). Aerosols may remain suspended in the air for a period of time, travel over a distance and may cause infection if inhaled (*Aerosol Generating Procedures (AGPs)* 2019).

## Aerosol emissions and coughing

The dichotomous definition of aerosols and droplets is an arbitrary one, based on droplet size rather than a formal measure of infection risk or transmission rate (Shiu *et al*. 2019, Bourouiba 2020). The boundary of distinction varies across the literature (Howard *et al*. 2020). In realistic contexts, respiratory droplet emissions from a cough or a sneeze form a complex cluster of droplets across a range of sizes and from different levels of the respiratory system, within a turbulent gas cloud, under forward momentum (Bourouiba *et al*. 2014). In contrast to laboratory‐based investigations of isolated droplets, the distance travelled by droplets emitted on a cough varies depending on a range of contextual factors: the patient's physiology, air flow currents, humidity and temperature (Zhu *et al*. 2006, Bourouiba *et al*. 2014). Other droplets may evaporate and remain suspended in the air for hours (Bourouiba *et al*. 2014). Coughing is an acknowledged source of aerosol droplet emissions (*Aerosol Generating Procedures (AGPs)* 2019, *Guidance: Transmission Characteristics and Principles of Infection Prevention and Control* 2020, *Infection Prevention and Control and Preparedness for COVID‐19 in Healthcare Settings. Second Update—31 March 2020* 2020, Greenhalgh 2020, Howard *et al*. 2020) and saliva droplets emitted during forceful coughing have been highlighted as an important route for virus transmission (Judson and Munster 2019, Zhu *et al*. 2006).

## Swallowing (dysphagia) assessment and coughing

Dysphagia assessment comprises several components, of which cough testing (voluntary cough), reflexive cough and swallowing trials with samples of fluid and food are of particular relevance to this report (Martino *et al*. 2004, Watts *et al*. 2016). Reflexive coughing, secondary to aspiration of food or fluid into the lungs, is a common but unpredictable occurrence inherent to specialist dysphagia assessment (Smith Hammond and Goldstein 2006). The resultant coughing may be forceful, prolonged and not easily suppressed (Mazzone 2005, Addington *et al*. 2008; expert opinion of the Advisory Group). In addition, many dysphagia assessment protocols include some form of testing the presence and strength of a patients’ voluntary cough as an indicator of their ability to protect their airway from aspiration of food or fluids (Watts *et al*. 2016). Undertaken by SLTs within 1 m of the patient, comprehensive dysphagia assessments are prolonged, lasting close to 10 min during which time coughing is tested or expected to occur (expert opinion of the Advisory Group). Ear, nose and throat (ENT) healthcare professionals have been reported to be at high risk of exposure and infection from COVID‐19 due to their close proximity to patients’ upper respiratory mucosa and interventional procedures that, similar to dysphagia assessments, induce cough (Givi *et al*. 2020, Lu *et al*. 2020). Given the proximity and prolonged exposure to frequent coughing during dysphagia assessment and strong theoretical risks, it is a reasonable assumption that SLTs are at a similarly high level of occupational risk of COVID‐19 infection.

## Dysphagia‐induced coughing and patients with COVID‐19

Clinically, many patients presenting with COVID‐19 (or suspected COVID‐19) and dysphagia are predisposed to coughing during dysphagia assessments as a result of their concomitant respiratory conditions: upper respiratory tract symptoms of the COVID‐19 infection, respiratory support requirements (Leder *et al*. 2015, Oomagari *et al*. 2015, Hori *et al*. 2016, Jaffe *et al*. 2018), reduced oxygen saturations (Steele and Cichero 2014), post‐acute respiratory distress syndrome (Brodsky *et al*. 2017) or other comorbidities (e.g., chronic obstructive pulmonary disorder; Cvejic *et al*. 2011). Dysphagia itself may have resulted in an aspiration pneumonia while oral, pharyngeal and laryngeal weakness (secondary to intubation, intensive care unit (ICU) acquired weakness or neurological conditions) reduces the patients’ ability to manage oral secretions and protect the airway (Scheel *et al*. 2016, Brodsky *et al*. 2017). Thus, patients presenting with COVID‐19 and dysphagia are predisposed to a heightened and more frequent coughing through aspiration of saliva, food or liquids.

## Aerosol‐generating procedures (AGPs)

AGPs are defined as ‘any medical and patient care procedure that results in the production of airborne particles (aerosols)’ (*Aerosol Generating Procedures (AGPs)*, 2020). At the time of writing, there is no consensus on a definitive list of healthcare procedures that are AGPs (Judson and Munster 2019) with variations in medical and care procedures considered to be AGPs across national policies (table 1) (Thompson *et al*. 2013, Shiu *et al*. 2019, *Use of PPE to Support Infection Prevention and Control Practice when Performing Aerosol Generating Procedures on Confirmed or Clinically Suspected COVID‐19 Cases in a Pandemic Situation* 2020). One recent review distinguished between AGPs that resulted in the creation or dispersion of aerosols and procedures that induced a patient to produce them (Judson and Munster 2019).

**Table 1:** Aerosol‐generating procedures (AGPs) by policy document

Procedure	UK Public Health England (COVID‐19 Personal Protective Equipment (PPE), 2020) and Health Protection Scotland (2019) (Aerosol Generating Procedures (AGPs), 2019)	New Zealand Ministry of Health (COVID‐19 Questions and Answers for Primary Health Care Workers, 2020)	Australian Government Department of Health (Interim Recommendations for the use of Personal Protective Equipment (PPE) during Hospital Care of People with Coronavirus Disease (COVID‐19), 2020)	Centre for Disease Control and Prevention (Healthcare Infection Prevention and Control FAQs for COVID‐19, 2020)	WHO IPC precaution recommendations (Modes of Transmission of Virus Causing COVID‐19: Implications for IPC Precaution Recommendations, 2020)
Intubation and extubation	Yes	Intubation only	Intubation only	Yes	Intubation only
Manual ventilation	Yes	Yes	Yes	Yes	Yes
Open suctioning	Yes			Yes	Yes
Tracheostomy and tracheostomy procedures	Yes	Yes	Yes		Yes
Bronchoscopy	Yes	Yes	Yes	Yes	Yes
Upper ENT airway procedures that involve suctioning	Yes				
Upper gastrointestinal endoscopy where there is open suctioning of the upper respiratory tract	Yes				
Cardiopulmonary resuscitation		Yes	Yes	Yes	Yes
Surgery and post‐mortem procedures involving high‐speed devices	Yes				
Some dental procedures (e.g., drilling)	Yes				
Non‐invasive ventilation, e.g., bi‐level positive airway pressure and continuous positive airway pressure	Yes	Yes	Yes	Yes	Yes
Disconnecting the patient from the ventilator					Yes
High‐frequency oscillating ventilation	Yes				
Induction of sputum	Yes	Yes		Yes	
High‐flow nasal oxygen	Added in 2020	Yes	Yes	State uncertain	
Turning the patient to prone position					Yes
Nebulizer treatment		Yes		State uncertain	Yes

Where possible, research evidence relating to acute respiratory infection transmission from patients to healthcare professionals in the context of specific healthcare procedures is used to identify AGP considered to be at high risk (*Infection Prevention and Control of Epidemic‐ and Pandemic‐Prone Acute Respiratory Infections in Health Care* 2014). The evidence base is limited, however, and biased in the selection of procedures investigated as sources of transmission (Tran *et al*. 2012, Thompson *et al*. 2013) later synthesized in reviews and meta‐analyses and, in turn, underpinning clinical recommendations.

Recent WHO guidelines on infection prevention and control (*Infection Prevention and Control of Epidemic‐ and Pandemic‐Prone Acute Respiratory Infections in Health Care* 2014), for example, refer to a systematic review in support of their classification of AGP and increased risk of SARS infection transmission (Tran *et al*. 2012). The systematic reviewers, however, used an earlier WHO‐generated list of AGPs to inform their review inclusion criteria. The reviewers highlight the lack of information available on procedures known to induce coughing and associated aerosol emissions and the possible risk of infection transmission associated with those procedures (Tran *et al*. 2012). Thus, as stated by the Centers for Disease Control and Prevention: ‘there is neither expert consensus, nor sufficient supporting data, to create a definitive and comprehensive list of AGPs for healthcare settings’ (*Healthcare Infection Prevention and Control FAQs for COVID‐19* 2020).

New AGPs continue to be identified through literature reviews of conflicting studies, theoretical risk of aerosol generation and expert consensus; non‐invasive ventilation and high flow nasal oxygen, for example, are two recent inclusions in UK health protection policy documents (*Aerosol Generating Procedures (AGPs)* 2019).

The research evidence to date on the risk of infection and transmission rate has focused on predefined AGPs. Within the systematic review of AGPs and risk of SARS transmission (Tran *et al*. 2012) all 10 included studies focused on intubation and ventilation procedures conducted by medical and/or nursing staff. Half did not appear to include SLTs; three of 10 studies focused on intubation and tracheostomy procedures only; two of 10 were specific to nursing or medical staff only. The remaining five studies (*n* = 1764 staff participants) referred to ‘other staff’ (*n* = 150), but no SLTs were described, nor was dysphagia assessment. Three studies recorded healthcare professionals’ contact with patient sputum and/or respiratory secretions, each reporting a significantly increased risk of infection. The quality of the primary research studies was poor, and the review syntheses were rated as low quality (Tran *et al*. 2012). As another example, a recent cluster randomized controlled trial evaluated the effectiveness of N95 respirators versus medical masks in reducing the risk of influenza transmission to healthcare professionals (Radonovich *et al*. 2019). The large trial took place across several US outpatient settings where dysphagia assessment is likely to be rare. SLTs were unreported amongst the 16 professionals recorded in the trial staff participant record form. The AGP recorded as undertaken by the staff included: intubation, respiratory/airway suctioning, nebulizer treatments and nasopharyngeal aspiration. While some research on AGP and risk of transmission exists, the evidence relating to dysphagia assessment and risk is absent, though this does not reflect an absence of risk.

## Procedures which induce forceful coughing

Induction of sputum following the administration of saline into the lungs, moistening and loosening respiratory secretions, shares an infection risk profile similar to dysphagia induced, prolonged and forceful coughing when food or fluid is aspirated into the lungs. The induction of sputum is currently considered an AGP (*Aerosol Generating Procedures (AGPs)* 2019). The recent European Centre for Disease Prevention and Control recommendations (*Infection Prevention and Control and Preparedness for COVID‐19 in Healthcare Settings. Second Update—31 March 2020* 2020) highlighted the risk of coughing or sneezing induced while collecting nasopharyngeal diagnostic respiratory samples, and the associated risk of aerosol production. Thus, there is consensus across the healthcare and infection control community that procedures known to induce prolonged and forceful coughing result in the production of aerosols which in turn place healthcare professionals at greater risk of infection. There is general agreement across the literature and guidelines that in such cases precautionary steps should be taken to reduce the risk of infection for healthcare professionals.

## Conclusions

We have presented evidence that forceful coughing generates aerosols and the emerging evidence which indicates that COVID‐19 is likely transmitted through aerosol (and other) routes. We described SLTs’ close and prolonged contact with forceful coughing, induced during standard dysphagia assessment procedures and why patients with COVID‐19 are likely to be at greater risk of coughing. We examined the criteria used to establish the current list of AGP and found a lack of consensus and a high risk of selection bias, focusing only on risk of infection based on previously identified AGPs. We highlighted evidence that suggests a greater risk of transmission and infection of healthcare professionals that experience frequent and repeated contact with patients’ respiratory secretions or sputum in the context of a novel virus, non‐immunity and high infectivity.

Amongst the limitations of our rapid review is the depth of searching and analysis of primary research findings that could be undertaken within a short timeframe. We were unable to identify definitive evidence linking dysphagia assessment to a higher risk of COVID‐19 transmission. However, it is important to note that we also failed to identify evidence that the procedure does not increase the risk of transmission. The strength of our rapid review includes the expert dysphagia authorship informed by a UK‐wide expert advisory group, a thorough search of published and grey literature, undertaken in a timely manner to address an urgent clinical question in the context of the COVID‐19 pandemic.

We presented strong theoretical reasons and underpinning empirical evidence to support our recommendation: that dysphagia assessment is considered an AGP. The following multidisciplinary professional associations and learned societies, which share our interest in this issue, are in support of our conclusions (see the Supplementary Materials):

- British Association of Parenteral and Enteral Nutrition.
- British Thoracic Society.
- British Association of Stroke Physicians.
- ENT‐UK.
- European Society of Swallowing Disorders.
- Intensive Care Society, National Tracheostomy Safety Project.
- UK Swallowing Research Group.

In the context of the available evidence and expert consensus, healthcare providers and infection control policy‐makers should take precautionary steps to reduce the risk of COVID‐19 transmission and infection while undertaking dysphagia procedures (*Infection Prevention and Control and Preparedness for COVID‐19 in Healthcare Settings. Second Update—31 March 2020* 2020). The safety of healthcare workers and expert consensus should prevail.

## Contributions

The review was prepared on behalf of the Royal College of Speech and Language Therapists (RCSLT) COVID‐19 Advisory Group. All co‐authors, L. B., C. M., S. W. and M. C. B., contributed equally to the drafting and review of the manuscript. The final draft was approved by the wider Advisory Group, as listed in the appendix.

## Supporting information

Supplementary Material

Supplementary Material

## Members of the Royal College of Speech and Language Therapists (RCSLT) COVID‐19 Advisory Group

Katherine Behenna, Lead for Head & Neck and Voice Disorders SLT Service, Nottingham University Hospitals Trust

Lee Bolton, Clinical Lead Speech & Language Therapist/Improvement Coach, Imperial Health Charity; Pre‐doctoral Research Fellow, Imperial College London

Grainne Brady, Clinical Lead Speech & Language Therapist Head & Neck, The Royal Marsden NHS Foundation Trust

Gemma Clunie, Clinical Specialist SLT (Airways/ENT) Imperial College Healthcare NHS Trust; HEE/NIHR Clinical Doctoral Fellow, Imperial College London

Dr Margaret Coffey, Clinical Service Lead, SLT (Head and Neck/ENT), Honorary Clinical Research Fellow, Imperial College London

Dr Hannah Crawford, Professional Head of Speech & Language Therapy, Tees, Esk & Wear Valleys NHS Foundation trust

Zoe Dalal, Team lead, Wellington Hospital

Pauline Downie, Professional Lead for Speech and Language Therapy, NHS Lanarkshire

Dr Roganie Govender, Consultant SLT & NIHR Clinical Lecturer, University College London Hospital, Head and neck Cancer Centre; Hon Senior Research Associate, University College London, Research Department of Behavioural Science & Health, Epidemiology & Public Health

Jemma Haines, Consultant Respiratory Speech & Language Therapist, NIHR Manchester BRC PhD Fellow & Service Lead for Manchester Airways Service, Manchester University NHS FT

Lauren Isaacs, Principal Speech & Language Therapist, Adult Community Speech & Language Therapy, Norfolk Community Health and Care NHS Trust

Gemma Jones, Clinical Lead SLT, Cardiff and Vale University Health Board; Highly Specialist SLT Critical Care, Cwm Taf Morgannwg University Health Board

Hannah Lewthwaite, Speech and Language Therapist, Clinical Expert Pathway Lead for Dysphagia, Solent NHS Trust

Julia McDowall, Clinical Lead Speech and Language Therapist (intensive care/neurosurgery), North Bristol NHS Trust

Dr Jackie McRae, Associate Professor and Director of Research, School of Allied Health, Midwifery and Social Care, University of London; Consultant Speech and Language Therapist, University College London Hospital

Claire Mills, Clinical Specialist Speech and Language Therapist in Critical Care; NIHR Clinical Doctoral Research Fellow, University of Leeds

Suzannah Partner, Clinical Lead Speech and Language Therapist, Community Neurorehabilitation, Central London Community Healthcare NHS Trust

Francesca Pepper, Clinical Lead Specialist, Adult Speech and Language Therapy, Hertfordshire division, Central London Community Healthcare

Professor Sue Pownall, Head of Speech and Language Therapy and Clinical Lead in Dysphagia, Academic Director Therapeutics & Palliative Directorate, Sheffield Teaching Hospital NHS Foundation Trust

Dr Justin Roe, Consultant and Joint Head, Department of Speech, Voice and Swallowing, The Royal Marsden NHS Foundation Trust; Clinical Service Lead, Speech & Language Therapy, National Centre for Airway Reconstruction/Department of Otolaryngology, Head and Neck Surgery, Imperial College Healthcare NHS Trust; Honorary Lecturer, Division of Surgery, Department of Surgery and Cancer, Imperial College London

Alex Stewart, NIHR Clinical Doctoral Research Fellow, Specialist Speech and Language Therapist, Great Ormond Street Hospital/UCL

Sarah Wallace, Consultant SLT (Critical care and dysphagia), Wythenshawe Hospital, Manchester University NHS Foundation Trust; Chair RCSLT Tracheostomy Clinical Excellence Network; NIHR Research Associate
