# Supplementary material for: Aerosol generating procedures, dysphagia assessment and COVID‐19: A rapid review
Source: Int J Lang Commun Disord. 2020 Jun 1;55(4):629–36. doi: 10.1111/1460-6984.12544 (PMC7300802; doi:10.1111/1460-6984.12544)
Supplement: Supplementary file 1 — Supplementary Material [file JLCD-55-629-s001.pdf]

Kamini Gadhok
Chief Executive
Royal College of Speech and Language Therapists
2 White Hart Yard
London
SE1 1NX

13 April, 2020

Dear Kamini

The intensive Care Society is a multi-professional membership organisation and a charity and some of our members are Speech and Language Therapists. We have been informed that the you are lobbying PHE to provide adequate personal protective equipment for speech and language therapists while they are conducting dysphagia assessments. We are writing to give you our support.

We recognise that there is currently a lack of research evidence directly looking at aerosol generation during dysphagia assessment. However, we know that these assessments often induce forceful and prolonged reflexive coughing. As such we support the SLT expert consensus that this is an aerosol generating procedure. This expert consensus should be sufficient for PHE to recognise and add dysphagia assessments to the list of AGPs.

Please contact us again if there is anything else we can do to support you and our SLT intensive care community.

Dr Ganesh Suntharalingam
President

Dr Sandy Mather
Chief Executive

CC: Claire Mills
Sarah Wallace

Intensive Care Society I Churchill House I 35 Red Lion Square I London I WC1R 4SG
T: +44 (0)20 7280 4350 E: info@ics.ac.uk W: www.ics.ac.uk

Patron: HRH The Princess Royal

Intensive Care Society. A company limited by Guarantee. Registered No: 2940178 (England) registered office as above
Registered as a Charity in England and Wales No: 1039236 and in Scotland No: SC040052

Acute ICU, Wythenshawe Hospital
Manchester University Hospital
Southmoor Road
Wythenshawe
Manchester, UK
M23 9LT

NTSP Ltd
National Tracheostomy Safety Project
www.tracheostomy.org.uk

Tel: 0161 291 6420
Fax: 0161 291 6421
Email: admin@tracheostomy.org.uk

Friday, 01 May 2020

Kamini Gadhok
Chief Executive Royal College of Speech and Language Therapists
2 White Hart Yard
London SE1 1NX

Letter in support of for the RCSLT position on dysphagia assessment with respect to the COVID-19 pandemic.

Dear Kamini

The National Tracheostomy Safety Project (NTSP) is a multi-professional organisation which collaborates with colleagues, patients and families around the world to improve tracheostomy care. As you are aware, Speech and Language Therapists (SLT) are closely involved in the front-line delivery of care to these patients as part of the multidisciplinary team and the importance of SLTs in managing these complex patients is emphasised by Mrs Sarah Wallace, RCSLT Tracheostomy lead, representing SLTs in the NTSP lead team.

We are aware that you are lobbying Public Health England to provide adequate personal protective equipment for speech and language therapists while they are conducting dysphagia assessments, which carry risks of aerosolization. This poses immediate and worrying risks for these vital front-line staff who are helping to manage and to rehabilitate patients during the COVID-19 pandemic. We are concerned about the risks to staff who may be inadequately protected and wish to offer our support. Currently tracheostomy insertion and removal are listed as aerosol-generating procedures, but not dysphagia assessment related to tracheostomy care. During such assessment, many of these patients are more likely to cough due to the nature of their condition and nearly all patients have excess oral and tracheal secretions. Whilst infectivity is difficult to quantify, most patients with tracheostomy or dysphagia who are seen by SLT during this pandemic will be recovering from critical illness, where prolonged detection of SARS-CoV-2 virus in respiratory and oral secretions has been clearly documented (see references below).

Accepting that the evidence base is weak across the board when it comes to aerosol-generating procedures, given the clear risks of viral transmission to staff associated with dysphagia assessment, the NTSP strongly supports the SLT expert consensus that this should be considered an aerosol generating procedure. Following on from this, it seems clear that staff should be protected with appropriate PPE to conduct these assessments in these high-risk encounters.

Please contact me if there is anything further that the NTSP can do to support you and our SLT colleagues.

Kind regards

With best wishes

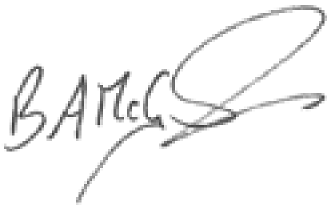

Dr Brendan A McGrath
National Clinical Advisor for Tracheostomy, NHS England.

Consultant in Anaesthesia & Intensive Care Medicine
University Hospital South Manchester
Chair National Tracheostomy Safety Project
European Lead Global Tracheostomy Collaborative

brendan.mcgrath@tracheostomy.org.uk

References

Zhao J, Yuan Q, Wang H, et al. Antibody responses to SARS-CoV-2 in patients of novel coronavirus disease 2019. Clin Infect Dis 2020.

Wölfel R, Corman VM, Guggemos W, et al. Virological assessment of hospitalized patients with COVID-2019. Nature 2020.

British Thoracic Society

Kamini Gadhok
Chief Executive
Royal College of Speech and Language Therapists
2 White Hart Yard
London
SE1 1NX

20 April, 2020
By email

Dear Ms Gadhok

As you know, the British Thoracic Society counts a number of Speech and Language Therapists among its members.

We are aware, through Jemma Haines, that the Royal College of Speech and Language Therapists is lobbying for the provision of adequate personal protective equipment for speech and language therapists while they are conducting dysphagia assessments (which carry risks of aerosolisation) and we are writing to confirm the Society's support for your request.

We hope that you will keep the Society informed of developments in this area. Thank you.

Kind regards

Professor J Bennett
Chair of Board of Trustees

Dr M Munavvar MD DNB FRCP
President

at The Royal College of Surgeons of England
35-43 Lincoln's Inn Fields
London WC2A 3PE

Tel: 020 7404 8373
Email: entuk@entuk.org Web: www.entuk.org

20 April 2020

Kamini Gadhok MBE
CEO
Royal College of Speech & Language Therapists
2 White Hart Yard
London SE1 1NX

Dear Kamini

Re: Letter in support of for the RCSLT position on dysphagia assessment with respect to the COVID-19 pandemic.

ENT UK is the professional membership body representing Ear Nose and Throat surgery, as well as its related specialties in the United Kingdom. As a registered charity, we support members at varying stages of their career in ENT, providing a clear vision for setting and delivering safe and effective service and training standards.

Our members work in close teamwork with speech and swallow therapy colleagues, often assessing and managing patients together in combined outpatient services. We fully sympathise and support your college's request to Public Health England that speech and language therapists be provided with full personal protective equipment to support appropriate dysphagia assessments.

Dysphagia assessments by their very nature involve the risk of triggering a protective cough, both at bed-side or endoscopy. Although research base is limited in relation to relative risk of aerosol generation during such assessments, there is multi-disciplinary expert consensus between ENT and speech therapy colleagues that dysphagia assessment should be considered high risk for aerosol generation and therefore high potential risk to professionals through droplet spread of Covid-19.

We write to express our organisation's full support in your request to Public Health England.

Yours sincerely,

B. Nirmal Kumar

Professor Nirmal Kumar
President ENT UK

Mr Taran Tatla
Honorary Secretary ENT UK

ENT UK trading as
British Academic Conference in Otolaryngology (BACO) and British Association of Otorhinolaryngology – Head & Neck Surgery (BAO-HNS)

Registered as a Company limited by Guarantee in England and Wales under Company No 06452601
Registered with the Charity Commission in England and Wales under Charity No 1125524

UK Swallow Research Group

Professor David G Smithard

BSc MBBS MD FRCP FRCLST(Hon)

Chair UKSRG

Queen Elizabeth Hospital

Stadium Road

Woolwich

SE18 4QH

20th April 2020

Kamini Gadhok MBE

CEO

Royal College Speech and Language Therapists

2 White Hart Yard

London SE1 1NX

Dear Kamini

The UK Swallowing Research Group is a multidisciplinary research based organisation. The members come from many different healthcare professional backgrounds, the largest being speech and language therapy. Speech and language therapists are vital to the management of patients with swallowing problems.

Assessments of a persons swallow involve a clinical assessment of the swallow, which may be followed by a fibre-optic endoscopic evaluation of the swallow or a Videofluoroscopy. Anyone of these swallowing assessments may trigger a cough reflex at any time, generating an aerosol of which the speech and language therapist will be in direct line. Endoscopy entails exposure to an aerosol during the procedure and whilst cleaning the endoscope.

Although there is little objective research around the clinical assessment, aerosol generation in a cough and endoscopy is accepted.

Given these facts, it is entirely appropriate that speech and language therapists should be provided with full PPE whilst undertaking these aerosol generating assessments. We would support the Royal College in their request to PHE.

UK Swallow Research Group

If we can be of any further assistance pleases get back in touch.

Yours sincerely

David G Smithard

Kamini Gadhok
Chief Executive
Royal College of Speech and Language Therapists
2 White Hart Yard
London
SE1 1NX

22 April, 2020
By email

Dear Ms Gadhok,

We are aware that the Royal College of Speech and Language Therapists is lobbying for the provision of adequate personal protective equipment for Speech and Language Therapists while they are conducting dysphagia assessments.

The ***European Society for Swallowing Disorders*** would like to express our support for the provision of adequate personal protective equipment for Speech and Language Therapists.

Dysphagia Assessment at the bedside includes Aerosol generating procedures. The clinical and scientific community is aware that these procedures consist of the highest risk of transmission of respiratory viruses, and use of enhanced respiratory protective equipment is indicated for health and social care workers performing or assisting in such procedures.

Assessment of Dysphagia is of vital importance in order to reduce the risk of malnutrition, dehydration, institutionalisation, poor medical care outcomes and aspiration pneumonia. If Dysphagia is not assessed consistently and timely, further threats to the health system are imposed (increasing the costs and prolonging hospital stay). Dysphagic symptoms not assessed may prove life threatening in specific patients.

We hope that you will keep the Society informed of developments in this area.
Thank you.

Kind regards,

Nathalie Rommel

President of ESSD

On behalf of the ESSD Board

BAPEN
Putting patients at the centre of good nutritional care
Registered Charity No.1023927

21st April 2020

Kamini Gadhok
Chief Executive
Royal College of Speech and Language Therapists
2 White Hart Yard
London
SE1 1NX

Dear Ms Gadhok

As you know the British Association of Parenteral and Enteral Nutrition (BAPEN) has close professional links with the Royal College of Speech and Language Therapists (RCSLT).

It has come to BAPEN's attention that RCSLT have approached Public Health England (PHE) to request that swallowing evaluations are designated as aerosol generating procedures (AGP).

BAPEN is aware that swallowing evaluations often elicit a cough response as a consequence of aspiration (food and liquid going down the wrong tube). On this basis, BAPEN is writing to confirm support for RCSLT's position that a swallow evaluation is recognised as an aerosol generating procedure. BAPEN has already written to PHE to request a change of status for Nasogastric tube insertion (NGT) to an AGP[1]. We would wish to point out that BAPEN has the support of many other professional bodies in its request for a revision of the designation of NGT to that of an AGP[1].

BAPEN believes that the evidence base for NGT being a non AGP is extremely lacking and furthermore, PHE has designated "cough induction" as an AGP[2,3]. Indeed, swallowing assessments bear many similarities to "cough induction" as defined by PHE. The science around coughing now indicates that coughing does generate aerosol as well as droplets.

The probability of a cough occurring during assessment of swallowing safety is so high that all such procedures should be considered as AGP during the Covid-19 crisis.

BAPEN also supports this stance as a means of ensuring the provision of personal protective equipment appropriate for an AGP as defined by PHE for Speech and Language Therapists and other healthcare professionals during the current COVID 19 pandemic.
BAPEN strongly supports the RCSLT submission to PHE.

Yours sincerely,

Dr Trevor Smith

President, BAPEN

1.BAPEN letter to PHE re NGT and AGP:
https://www.bapen.org.uk/pdfs/covid-19/bapen-letter-to-public-health-england.pdf.

2. BAPEN overview of NGT as AGP during Covid-19:
https://www.bapen.org.uk/pdfs/covid-19/ngt-and-agp-and-ppe-15-04-20.pdf

3. BAPEN NGT safety in Covid-19:
https://www.bapen.org.uk/pdfs/covid-19/covid-19-and-enteral-tube-feeding-safety-16-04-20.pdf

Kamini Gadhok
Chief Executive
Royal College of Speech and Language Therapists
2 White Hart Yard
London
SE1 1NX
via email to louise.borjes@rcslt.org

23 April, 2020

Dear Kamini

Support for the RCSLT position on adequate personal protective equipment for swallow assessments during the COVID-19 pandemic

We are writing on behalf of the British Association of Stroke Physicians (BASP), which is a registered charity and professional association representing clinicians who care for patients with stroke in the British Isles. We support the RCSLT request for adequate personal protective equipment for healthcare professionals conducting swallow assessments, which carry risks of aerosolisation and put these healthcare workers at further risk during the COVID-19 pandemic.

BASP believes that speech and language therapists have an important role, based on good evidence, to not only improve communication for stroke survivors with language difficulties (aphasia),[i] but also to assess patients' swallowing so that patients with dysphagia can benefit from early nasogastric tube feeding, which can improve outcome.[ii] This is why early assessments of swallowing are part of standard practice for all of our patients with stroke.

Speech and language therapists and nurses provide front line assessments of swallowing and recommend insertion of nasogastric tubes, both of which carry a risk of aerosolisation of the contents of patients' upper airways. We recognise that there is currently a lack of research evidence directly looking at aerosol generation during these procedures, but we know that these assessments often induce forceful and prolonged reflexive coughing. This puts the healthcare workers conducting these procedures at further risk during the COVID-19 pandemic, when patients with stroke (who are usually older with multiple co-morbidities) are at greater risk of COVID-19.

Therefore, we support the Royal College of Speech and Language Therapists expert consensus that conducting a swallow assessment is an aerosol generating procedure and requires the commensurate personal protective equipment for healthcare professionals conducting these assessments during the COVID-19 pandemic. We believe that the same applies to nasogastric tube insertion.

We therefore support the Royal College of Speech and Language Therapists in requesting that swallow assessments and nasogastric tube insertion are added to the list of aerosol generating procedures to ensure appropriate personal protective equipment guidance is recommended for the healthcare workers conducting these procedures.

Yours sincerely,

Rustam Al-Shahi Salman

Prof. Rustam Al-Shahi Salman
President of BASP
Honorary consultant neurologist

Dr. Michelle Dharmasiri
Chair of BASP clinical standards committee
Consultant stroke physician

[i] Brady MC, Kelly H, Godwin J, Enderby P, Campbell P. Speech and language therapy for aphasia following stroke. Cochrane Database of Systematic Reviews 2016, Issue 6. Art. No.: CD000425. DOI: 10.1002/14651858.CD000425.pub4.

[ii] Dennis MS, Lewis SC, Warlow C; FOOD Trial Collaboration. Effect of timing and method of enteral tube feeding for dysphagic stroke patients (FOOD): a multicentre randomised controlled trial. Lancet. 2005;365(9461):764–772. doi:10.1016/S0140-6736(05)17983-5

British Association of Stroke Physicians, Blackburn House, Redhouse Road, Bathgate, EH47 7AQ
Tel: 01506 292046 **Email:**stacy.martin@fitwise.co.uk **Website:**www.basp.ac.uk
