# Supplementary material for: Aerosol generating procedures, dysphagia assessment and COVID‐19: A rapid review
Source: Int J Lang Commun Disord. 2020 Jun 1;55(4):629–36. doi: 10.1111/1460-6984.12544 (PMC7300802; doi:10.1111/1460-6984.12544)
Supplement: Supplementary file 2 — Supplementary Material [file JLCD-55-629-s002.docx]

|  | **Medline Search Strategy** |
| --- | --- |
| 1 | ((aerosol* or droplet* or infection* or infectious or disease*) adj6 (generat* or produc* or respirable range* or dispers* or transmission or transmitted or transmit or spread* or disseminat*)).ti,ab. |
| 2 | ((virus* or viral) adj3 (respirable range* or dispers* or transmission or transmitted or transmit or spread* or disseminat*)).ti,ab. |
| 3 | Infectious Disease Transmission, Patient-to-Professional/ |
| 4 | Disease Transmission, Infectious/ |
| 5 | Infectious Disease Transmission, Professional-to-Patient/ |
| 6 | Occupational Exposure/ |
| 7 | Air Microbiology/ |
| 8 | 1 or 2 or 3 or 4 or 5 or 6 or 7 |
| 9 | Infection Control/ |
| 10 | ((infection* or disease*) adj3 (prevent* or control* or limit* or protect*)).ti,ab. |
| 11 | (PPE or RPE).ti,ab. |
| 12 | (protect* adj1 equipment*).ti,ab. |
| 13 | 9 or 10 or 11 or 12 |
| 14 | (health care worker* or healthcare worker* or health care provider* or healthcare provider* or physiotherapist* or dentist* or nurse* or doctor* or physician* or health personnel or medical personnel or hospital personnel or hospital worker* or staff or healthcare professional* or health care professional* or care giver* or caregiver* or paramedic* or therapist*).ti,ab. |
| 15 | therapist*.ti,ab. |
| 16 | 13 and 15 |
| 17 | ((speech or language) adj1 (therap* or practitioner* or professional*)).ti,ab. |
| 18 | 8 or 13 |
| 19 | 17 and 18 |
| 20 | (swallow* or dysphagia or deglutition).ti,ab. |
| 21 | 18 and 20 |
| 22 | 14 and 21 |
| 23 | exp coronavirus/ |
| 24 | ((corona* or corono*) adj1 (virus* or viral* or virinae*)).ti,ab,kw. |
| 25 | (coronavirus* or coronovirus* or coronavirinae* or Coronavirus* or Coronovirus* or Wuhan* or Hubei* or Huanan or "2019-nCoV" or 2019nCoV or nCoV2019 or "nCoV-2019" or "COVID-19" or COVID19 or "CORVID-19" or CORVID19 or "WN-CoV" or WNCoV or "HCoV-19" or HCoV19 or CoV or "2019 novel*" or Ncov or "n-cov" or "SARS-CoV-2" or "SARSCoV-2" or "SARSCoV2" or "SARS-CoV2" or SARSCov19 or "SARS-Cov19" or "SARSCov-19" or "SARS-Cov-19" or Ncovor or Ncorona* or Ncorono* or NcovWuhan* or NcovHubei* or NcovChina* or NcovChinese*).ti,ab,kw. |
| 26 | "severe acute respiratory syndrome*".ti,ab,kw. |
| 27 | 23 or 24 or 25 or 26 |
| 28 | 17 and 27 |
| 29 | 20 and 27 |
| 30 | 13 and 14 and 27 |
